# Lessons from using SALAMA (DIGIT HCM—health campaign management platform) to implement and optimize seasonal malaria chemoprevention in Nampula, Mozambique

**DOI:** 10.1093/oodh/oqaf036

**Published:** 2026-01-06

**Authors:** Abdul Mussa, Maria Rodrigues, Sonia M Enosse, Louise Cook, Liberty Bunce

**Affiliations:** Malaria Consortium (Mozambique), Av. Lucas Elias Kumato nr. 118, Bairro da Sommershield, Maputo, Mozambique; Malaria Consortium (Mozambique), Av. Lucas Elias Kumato nr. 118, Bairro da Sommershield, Maputo, Mozambique; Malaria Consortium (Mozambique), Av. Lucas Elias Kumato nr. 118, Bairro da Sommershield, Maputo, Mozambique; Malaria Consortium (Uganda), Plot 25, Upper Naguru East Road, Naguru P.O. Box 8045, Kampala, Uganda; Malaria Consortium (UK), The Green House, 244-254 Cambridge Heath Road, London, E2 9DA, London, UK

**Keywords:** malaria, digital health, data use, community based health information systems, Nampula province, northern Mozambique

## Abstract

Mozambique has the fifth highest malaria burden globally, with children under five most affected. To reduce this impact, Mozambique adopted seasonal malaria chemoprevention as a national strategy. In 2024, the National Malaria Control Program, in collaboration with Malaria Consortium and eGovernments Foundation, transitioned from paper-based to digital data collection using the DIGIT Health Campaign Management platform. We describe the digitalization process from planning to implementation and document the lessons. A cascade training and real-time dashboards facilitated data monitoring, stock tracking and supervision. Key results included improved sulfadoxine–pyrimethamine plus amodiaquine administration, enhanced data use for daily decision-making and early identification of outliers. For example, in Moma district sulfadoxine–pyrimethamine plus amodiaquine wastage exceeded 10% on the first day—unusual wastage rates are defined as >5%—prompting immediate targeted supervision which reduced wastage on subsequent days. Challenges including device power constraints, internet connectivity and local technical capacity, which were addressed through training and on-the-ground technical support. The digital approach improved campaign efficiency, transparency, responsiveness and supervision quality. Administrative coverage rates (75–109%) were more consistent with survey-based household coverage (~74%) in the digital campaign than the paper-based campaign which were 93–105% and ~79%, respectively. Importantly, the digital approach allowed for the visualization of the proportion of first-day doses recorded digitally in real time and the direct observation of therapy adherence, an important indicator of seasonal malaria chemoprevention campaign quality. This experience highlights how digital innovations, when well-coordinated and adapted to local contexts, can enhance malaria prevention and provide a case study to inform the scaling digital health interventions across public health programmes in Mozambique.

## INTRODUCTION

Malaria continues to be a significant public health challenge in Mozambique. In 2023, the country ranked fifth highest for malaria burden, accounting for ~4% of global malaria cases [1]. To address this, Mozambique implements several malaria control strategies, including insecticide-treated net distributions (ITNs), indoor residual spraying, perennial malaria chemoprevention and seasonal malaria chemoprevention (SMC) [2–6].

In Mozambique, SMC is delivered through community-based campaigns conducted over 4 monthly cycles during the rainy season. SMC involves intermittent administration of sulfadoxine–pyrimethamine and amodiaquine (SPAQ) to asymptomatic children aged 3–59 months [6]. The delivery system involves a complex logistics chain and substantial data collection demands across community, health facility, district and provincial levels. Real-time supervision includes monitoring appropriate dosing, delivery of information to caregivers and SMC administration documentation, ensures quality control [6].

Previous SMC campaigns have used paper-based data collection methods. However, these present challenges—including data inaccuracies and delays in data consolidation—which limit timely decision making and campaign effectiveness. Digital data collection tools improve data quality and allow real-time supervision and faster decision-making and improve resource use [4].

Digitalization is gaining interest for health campaign planning and implementation, and many national malaria programmes have incorporated digital tools and approaches within SMC campaigns, ranging from small pilots using digital tools specifically for planning or monitoring and evaluation, to larger-scale implementations with digital tools replacing all data collection [7].

The Mozambique National Malaria Control Program (NMCP) has previously used digital tools to digitize ITN campaigns. Recognizing the benefits of digitalization, the NMCP transitioned from paper-based to digital data collection for the 2023–2024 SMC campaign to improve data quality and reduce time between data collection and decision-making. The approach was designed to support data reuse to optimize campaign investments, improve data storage and data flow from community to national levels, and ensure integration with national health information systems [8]. This paper presents a case study of the SMC digitalization process, documenting campaign activities and lessons.

## METHODS

This manuscript presents a case study of operational insights, implementation experience and lessons learned from the digitalization of Mozambique’s 2023–2024 SMC campaign, so the headings of the sections are structured to reflect the real-world workflow of the SMC campaign.

### Paper-based campaign process

In paper-based SMC campaigns community distributors conduct door-to-door visits carrying printed tally sheets and referral forms which are used to record demographic eligibility information. Laminated guidelines or memory recall are used to screen for eligibility. Adverse events are recorded on paper referral slips.

Each day, distributors submit forms to health facilities, where staff manually compile summaries. Supervisors use paper checklists during spot checks and at the end of each cycle, health facilities aggregate data for cycle reports which are physically delivered to district health offices, where they are compiled and sent to provincial teams.

This process is vulnerable to data loss, transcription errors and double counting. Supervisors have limited oversight of progress, delaying corrective actions. Stock management also uses paper-based logs. Overall, the system limits timely data verification, real-time supervision and responsive campaign management.

### Planning for 2023–2024 digitalized campaign

Campaign microplanning used historical administrative data from previous SMC campaigns at health area level, with a 3.5% growth factor applied, reflecting Nampula province’s annual population growth. These figures were compared with census projections, and the higher estimate was used as a denominator for planning. Additionally, a comprehensive plan was developed estimating the human, logistical and financial resource needs for the campaign.

### Tool design

In September 2023, a meeting was held with the NMCP, Malaria Consortium and eGov to define the scope and scale of SMC digitalization in Mozambique. This included establishing a SMC coordination group and defining roles and responsibilities for programme coordination and decision making, and a national technical working group (TWG) to discuss operational and digital aspects of SMC implementation. To inform the development of the digital platform, the coordination group and TWG met bi-weekly and weekly, respectively. These meetings ensured SMC forms for stock management, registration and distribution, referral, pharmacovigilance, and supervision and processes and programme needs were reflected on the SALAMA platform. The platform incorporated two different components—an android application and web dashboard.

During tool design, the WHO Digital Health Interventions Classification Framework was reviewed to ensure the development process considered data collection requirements for SMC campaigns as well as user needs (Table 1). This table illustrates how SALAMA’s practical features aligned with the WHO Digital Health Interventions Classification Framework. This approach helps clarify how global digital health principles were operationalized in Mozambique’s 2023–2024 SMC campaign, as well as which areas remain underdeveloped or unsupported.

**Table 1:** WHO Digital Health Intervention category versus SALAMA features

WHO Digital Health Intervention category	WHO subcategory/domain	Corresponding SALAMA functionality	Description
Clients	Targeted communication to persons	Not implemented	SMS reminders for Days 2–3 not included
Health care providers	Provider decision support	Age-based inclusion validation; dose selection	But lacks automated exclusion logic
Health care providers	Provider-to-provider communication	Real-time dashboard syncing	Limited to supervisors; no Community Health Workers (CHW)-to-Health Facility (HF) feedback loop
Health system managers	Supply chain management	SPAQ stock entry, exit, inventory	Partially used; some incomplete entries
Health system managers	Public health event notification	SPAQ wastage and adverse events alerts	Used for supervision feedback
Data services	Data collection	Household and SPAQ records	Via mobile app
Data services	Data aggregation	Aggregated at dashboard level	Real-time sync
Data services	Data visualization	Web and app dashboards	Used for daily decision-making
Data services	Data quality assurance	Age/dose validation	No duplication/error detection
Data services	Data storage & transmission	Secure offline sync	Sync failures early in the campaign

### Users and use cases

**Figure 1:**
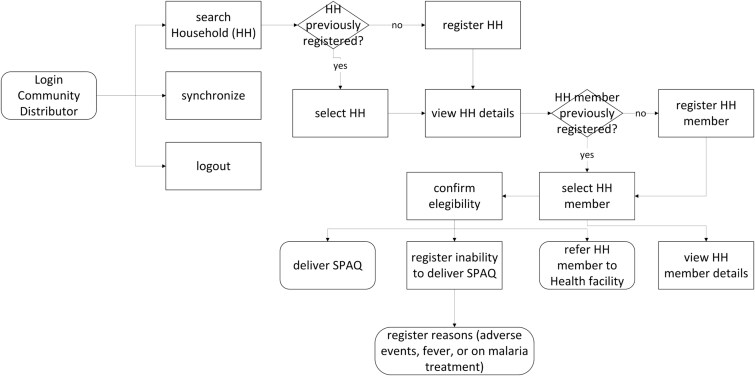
SALAMA community distributors workflow

**Figure 2:**
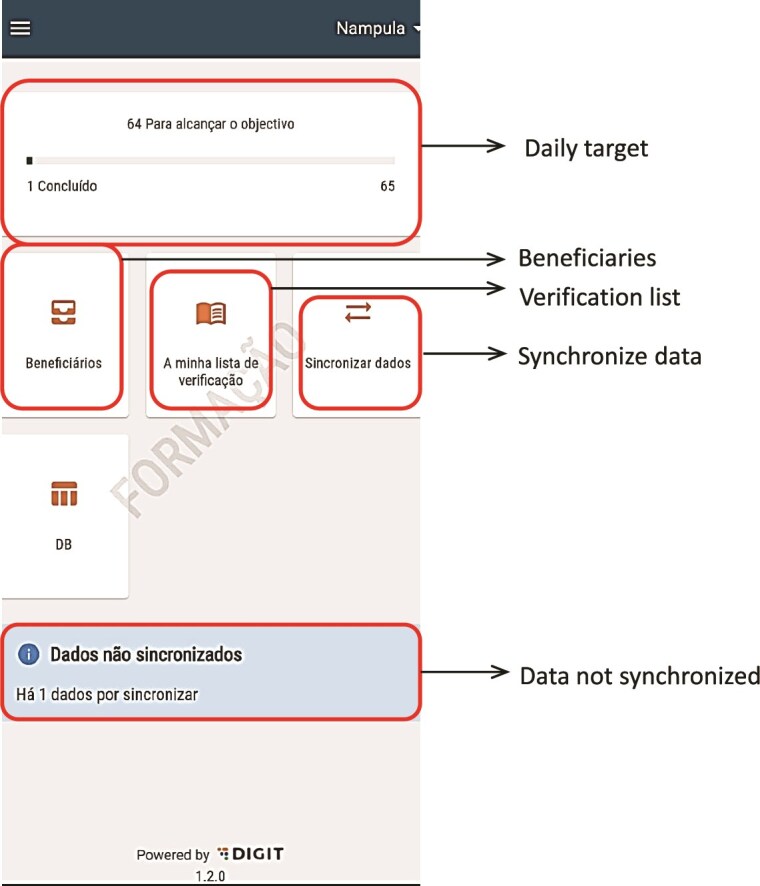
SALAMA app—community distributor profile—main page, after login

**Figure 3:**
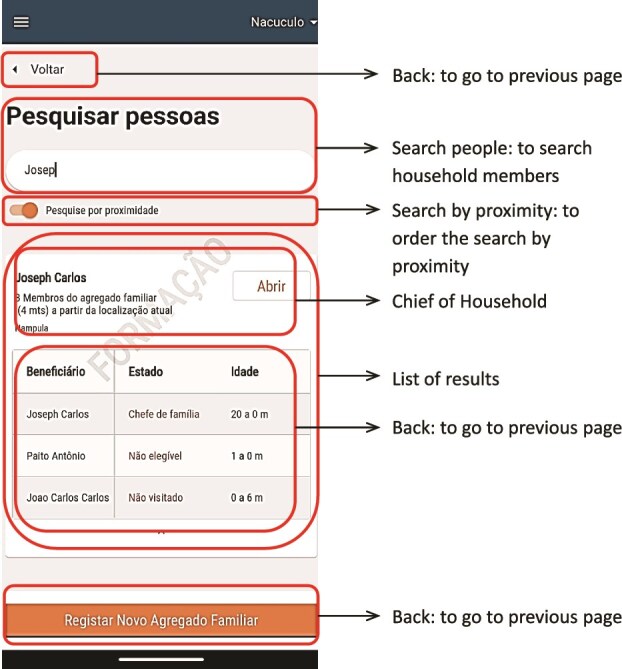
SALAMA app—community distributor profile after clicking on the beneficiaries button—for search and add beneficiaries page

**Figure 4:**
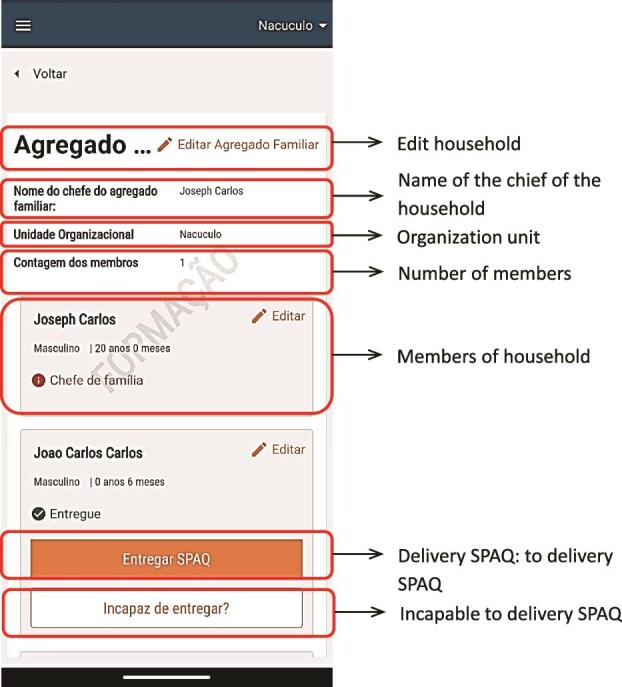
SALAMA app—community distributor profile—household details page

**Figure 5:**
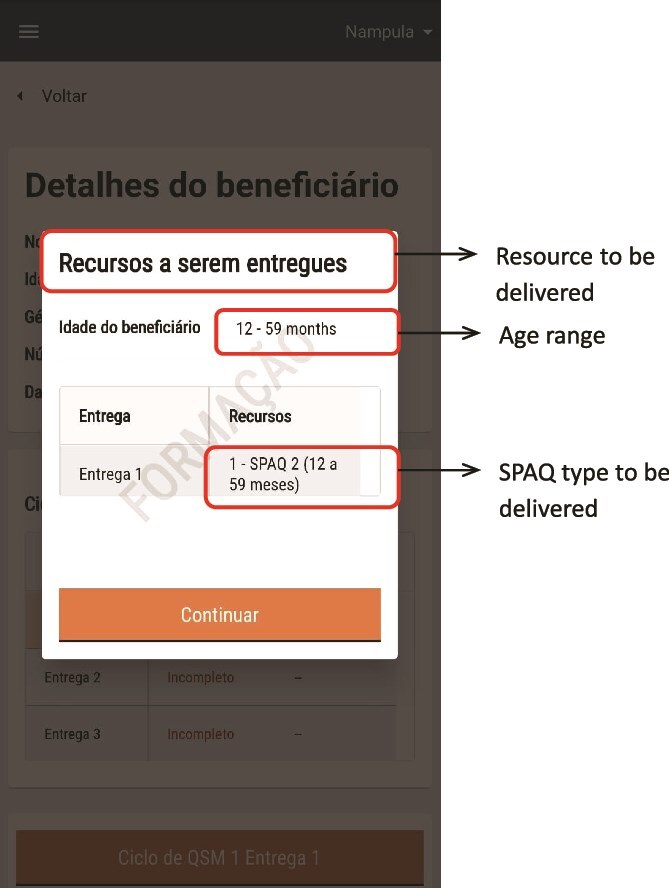
SALAMA app—community distributor profile—SPAQ delivery

**Figure 6:**
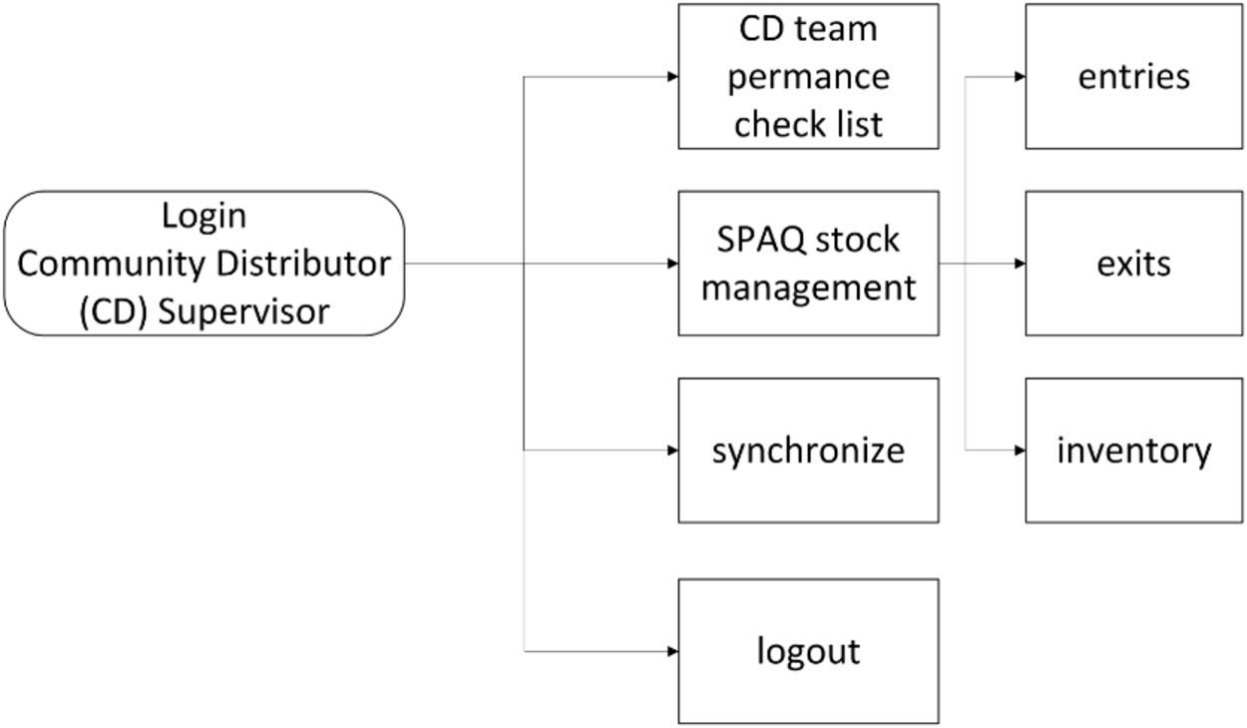
SALAMA community distributor supervisor workflow

**Figure 7:**
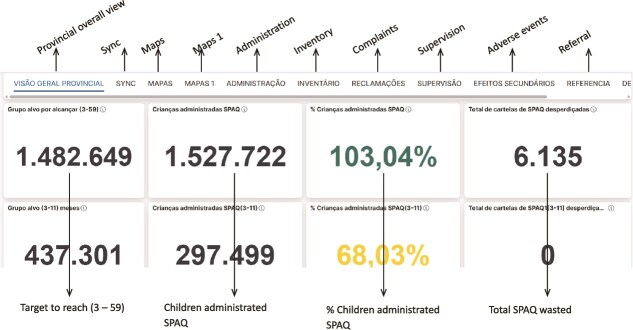
SALAMA overall dashboard, for cycle 4

There were two main users of the SALAMA android application (Table 2)—community distributors and community distributor supervisors. Distributors used the application in the field to register households and eligible children; record SPAQ administration, along with recording any children not receiving SMC, for example children currently unwell with malaria; record adverse events or side effects; record wasted, lost or damaged SPAQ to support stock management processes; and log issues and requests for technical support (see workflow in Fig. 1). To do this, distributers were first required to log in to the SALAMA application main page and select the ‘beneficiaries’ icon (Fig. 2). Following this, distributors could search for and select specific households and household members if they are already registered on the application or alternatively register a new household (Fig. 3). Only after selecting a household and household member (eligible child) was a distributor able to proceed to record SPAQ delivered or the inability to delivery SPAQ (such as for fever, adverse events and child requiring referral to health facility or APS) (Fig. 4). When registering the SPAQ delivery the SALAMA platform helps the community distributor select the correct SPAQ dosage, determined by the age of the registered household member (Fig. 5). Community distributor supervisors mainly engaged with the SALAMA application through completing digital checklists to verify community distributors’ performance, and SPAQ stock levels (Fig. 6). Supervisors from district and provincial levels used the application to review data synced in the platform, enabling them to monitor team progress and provide support based on performance. The SALAMA dashboard (Fig. 7) was designed for use by health officials from provincial and district levels, malaria programme staff from national, provincial and district level and partners—Malaria Consortium and eGov. The interactive dashboards and customized reports equipped these stakeholders with campaign performance data, enabling campaign monitoring and implementation informed by near real-time data.

**Table 2:** Training sessions

Training	Number of days per session	Number of sessions
Master ToT	3	1
Provincial ToT	3	1
Regional ToT	3	5
District ToT	3	23
Health facility training	2	600
Total	14	630

#### Dashboard

Between 22 January and 2 February 2024, the TWG held daily meetings to validate the SALAMA dashboard (Fig. 7) and customized reports. During the meeting, the TWG discussed the indicators, tables, maps and graphs.

### Location

**Figure 8:**
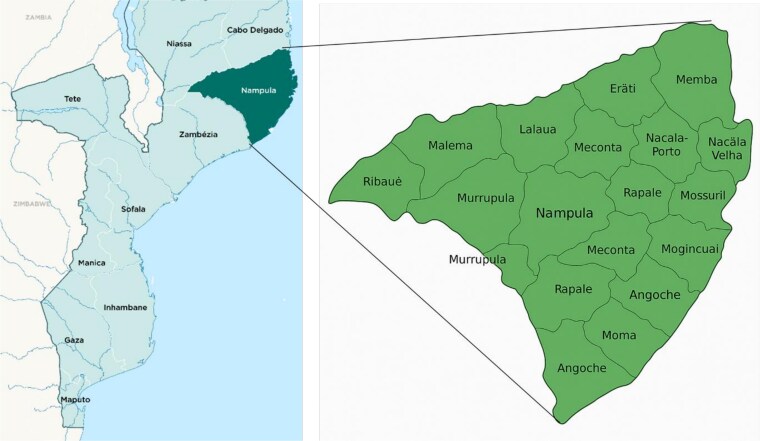
Location of Nampula province, Mozambique

The project was conducted in all 23 districts of Nampula province in Mozambique (Fig. 8).

### Testing

In November 2023, a two-day user acceptability testing session was conducted to identify bugs and usability issues, and to collect user insights. The testing was held in one of Nampula’s district health facilities, and involved the simulation of registering households, household’s members, SPAQ delivery, adverse events and referrals on the application. The testing exercise included 15 participants including national, provincial, health facility staff, community distributors and supervisors and the partner organizations. The user acceptability test achieved a 100% pass rate with no bugs identified. However, 14 change requests were addressed in the final release of the application, and 8 programmatic observations were resolved by updating standard operating procedures and improving training materials.

### Training

Once the platform testing had been completed, a master training of trainers (ToT) was delivered covering the SALAMA platform and application workflows. This was followed by a cascade of trainings: provincial ToT, regional ToT across introduction districts, district-level ToTs in Nampula’s districts, and training for community distributor supervisors, community distributors and community health workers at health facilities. The number of sessions per facility varied based on the number of local trainers and the local strategy.

Training covered the overall SMC campaign and use of the digital tool to ensure participants could fully integrate digital workflows into campaign activities. Sessions combined theory with practice, including topics such as SMC objectives, eligibility criteria, SPAQ administration, referral and adverse event management, communication with caregivers, safeguarding and use of smartphones and the SALAMA application for registration, stock taking, supervision and pharmacovigilance reporting. Role-plays and live data-entry exercises helped participants to practice daily workflows, supported by printed job-aids, building practical skills for using the digital tools during implementation and for supervision.

### Implementation

#### Device preparation

Prior to the campaign, and following each cycle, a device readiness process took place to check device performance and charging, to register the phone details including their condition, mapping the intended location for the device to be used within the campaign, platform deployment, accessories testing and synchronization.

#### SPAQ administration

Between February and May 2024, community distributors delivered SPAQ through four SMC cycles, following a door-to-door approach and collecting data using SALAMA (Fig. 1). During each cycle, community distributors visited households directly distributed SPAQ to eligible children through Directly Observed Treatment (DOT), and provided the child’s caregiver with subsequent dosage for Day 2 and Day 3 for the caregiver to administer to the child. The distribution process followed WHO recommendations described in detail in the WHO SMC field guide [6].

#### Monitoring and evaluation

**Figure 9:**
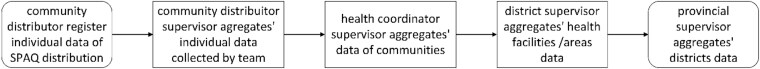
SMC paper-based M&E data collection & aggregation

In previous paper-based campaigns, data management involved the completion of child registration card followed by tally sheets by community distributors, the aggregation of individual data at each community by the distributors’ supervisors, the aggregation of all community data by the health facility coordinator, then all health areas’ data by the district supervisors and finally all districts’ data by provincial supervisors (Fig. 9). This typically led to a lack of data completeness for end-of-day data discussions. Other monitoring and evaluation activities based on data were conducted at least 1 day after data discussion meetings.

**Figure 10:**
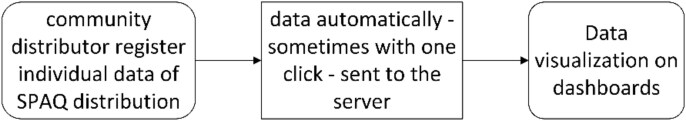
SMC SALAMA data collection, aggregation and visualization

With digitalization, data aggregation was completed automatically, sent to the server either automatically or through a one-click data synchronization function and visualized through online interactive dashboards (Fig. 10). Other monitoring and evaluation activities were performed by supervisors from national to district levels. The SALAMA dashboard and customized reports were used to monitor data quality and team performance for key performance indicators—including numbers/percentage of SPAQ administrated, number of referrals, number of adverse events, number of users who synced and more.

#### Data review and use

Data discussions took place daily using the data inputted into SALAMA and synchronized with the web-based dashboard. The discussions involved medical chiefs, public health department chiefs, health directors from provincial and district levels, malaria programme staff from national, provincial and district level and partners—Malaria Consortium and eGov. During the meetings, the group reviewed and discussed the performance of key indicators on the SALAMA dashboard with district level officials to enable data-informed decisions on changes needed to improve campaign performance and support campaign planning.

#### Supervision

Supervision and implementation support activities were tailored and conducted by supervisors of national (11), provincial (39), district (89), health facility (245) and community (975) levels to support both programmatic and digital components, based on data discussions. Programmatic support was provided by programme supervisors, including malaria programme managers and monitoring and evaluation technicians. Digital supervisors included IT and digital health technicians and monitoring and evaluation technicians. Central, provincial and district technical staff provided technical support to field teams and responded to issues reported to the SALAMA helpdesk and other coordination mechanisms, including WhatsApp.

### Successes and challenges

#### Management

Strong leadership from the NMCP at central and provincial levels enabled effective coordination and real-time data use. This was reinforced by strong data ownership at the provincial level. The TWG played a crucial role in digital tool design, ensuring key application functionalities were operational—including reliable offline data collection—and all campaign needs were met. However, delays in TWG formation and overlapping activities within the NMCP affected workflow efficiency, unrelated to the digital tool itself. The high number of features requested also delayed delivery with some features—notably those for pharmacovigilance—remaining incomplete at rollout.

Unforeseen challenges arose, which required deviations from planned activities and led to increased costs and unexpected logistical needs. The number of provincial supervisors almost doubled (23–40) requiring more vehicles (56–70). Additional costs included transporting devices from districts to Nampula city for readiness activities and procuring over 1000 extra data bundles (~3.20 USD each per cycle). These planning issues led to a 2-day delay launching the first SMC cycle.

#### Training

Training sessions (Table 2) were successfully conducted at all levels, with trainers and supervisors effectively delivering content, showing strong mobilization during the digital transition. However, several challenges were observed, including some features—such as supervision forms—not being included in the training application at the beginning of the master training, weak internet bandwidth in provincial training sites and the absence of printed user manuals for reference. These issues were further compounded by low digital literacy among some implementers.

#### SPAQ distribution

Campaign data showed improvements in community distributor’s digital skills across campaign cycles, with SPAQ distribution completion rates rising from 75.33% in cycle 1 to 81.25% in cycle 2, 109.62% in cycle 3 and 103.04% in cycle 4. Underreporting in cycle 1 was attributed in part to synchronization issues, rather than user performance. Adjustments were made to synchronization in cycle 1 which resulted in improvements 75.94% on cycle 1 Day 1, 80.03% on Day 2, 81.91% on Day 3 and 83.77% on Day 4. Operational challenges included duplicate household registration and repeat SPAQ administration entries for the same child within one cycle, and incorrect wastage reporting. Additionally, in the first cycles age validation mechanisms built into SALAMA prevented the recording of SPAQ administration to children who were eligible to receive SMC at the beginning of the campaign after they ‘aged out’ mid-campaign, despite guidance to include these children throughout the full campaign. This age validation mechanism was removed later in the campaign to enable these children to continue to receive SPAQ.

Compared to the 2022–2023 paper-based campaign (93–105% coverage), the 2023–2024 [9] digital campaign (75–109%) reflects improved data quality through in-app age and dose validation checks [10]. However, the system still revealed differences between districts and cycles, helping supervisors identify local performance variations. End-of-round survey data confirmed comparable overall coverage (2022–2023: ~79.2%; 2023–2024: ~74.05%) in cycle 4, but the digital approach, enabled precise monitoring of Day 1 SPAQ administration (70.65%) which was not possible through the paper-based approach. Pharmacovigilance reporting also improved, but lacked feedback loops from health facilities, limiting follow-up of referred patients.

Technical support was strengthened by an active helpdesk team providing virtual and field-based assistance. However, tracking issues reported through WhatsApp proved challenging, and there was limited technical capacity to resolve and monitor problems. This was enhanced from the second cycle, by training district monitoring and evaluation staff to resolve digital technical issues.

#### Supervision

The multi-tiered supervision structure played a vital role in campaign quality assurance, with national, provincial and district supervisors conducting real-time monitoring and evaluation. A total of 238 supervisions were conducted, 131 from district officials, 83 by central and provincial staff and 24 from Malaria Consortium staff were conducted. However, community distributor supervisor’s and health facility coordinator’s SALAMA profiles didn’t include a feature to view teams’ performance.

#### Monitoring and evaluation

The campaign made effective use of the SALAMA dashboard to track key indicators in real time, including SPAQ administration, synchronization performance, stock inventory, referrals and suspected adverse events. This enabled real-time performance monitoring and outlier identification. According to data from previous SMC campaigns, SPAQ wastage usually does not exceed 5%. However, in this campaign, it was detected through the SALAMA dashboards that several districts reported high wastage on the first day of cycle 1—e.g., Moma district reported the highest wastage (SPAQ1: 10.60%; SPAQ2: 10.15%), with these high wastage levels later confirmed during field visits that was a typo. Early identification led to mentoring during supportive supervision. However, challenges persisted in identifying data entry errors, tracking absentee households and assessing supervision effectiveness.

#### Data review and use

Daily data review meetings were held throughout the SPAQ distribution. These sessions served as platforms for sharing the status of key indicators, such as data synchronization, SPAQ administration performance, stock inventory levels, referrals, suspected adverse events and related outliers. Experiences from across Nampula were shared including successes, challenges and mitigation strategies. District and provincial action plans were created to improve campaign performance in real-time. However, poor battery life and internet connectivity in remote areas, which prevented data synchronization, compromised data reliability and completeness.

## DISCUSSION

The digital approach improved (i) efficiency, by enabling data-informed prioritization and tailoring of resources including supervisors, stock and technical support; (ii) transparency, through real-time data available through interactive dashboards and customisable reports connecting district to individual distributor level; (iii) responsiveness, by using near-real time data to identify anomalies and respond with targeted solutions and (iv) supervision quality, using digital checklists to improve consistency and quality of supervision.

### Management

Strong leadership and coordination at national and provincial levels enabled effective real-time data use. Early TWG engagement, structured coordination and realistic development timelines supported optimizing application performance and usability. However, unexpected increases in supervisors and logistical needs highlighted gaps in planning. Similar challenges occurred during Nigeria’s polio vaccination campaign, where insufficient community engagement disrupted service delivery [11]. Future campaigns should embed robust early operational readiness assessments to anticipate and mitigate challenges.

### Training

Training reached over 14 500 implementers; however, despite targeted training combining theory with hands-on practice and smartphone basics, digital literacy gaps affected initial performance. Rwanda’s Human Papilloma Virus (HPV) vaccination rollout showed continuous device-use reinforcement and early adaptation to digital training improved field performance and data quality [12]. Future campaigns could incorporate additional training to reinforce learning, such as digital refreshers, self-guided materials or bring your own device strategies to increase usability and system fluency. However, this assumes everyone with a smartphone is already a confident user.

### SPAQ distribution

Digital skills improved over the four cycles, reflected by increasing SPAQ administration rates. However, duplicate household registrations and incorrect SPAQ wastage reporting remained issues. Ethiopia’s measles outbreak response demonstrated integrating automatic anomaly detection into dashboards can reduce errors and improve data quality [13]. Implementing real-time validation features within SALAMA would strengthen data integrity. In addition, pharmacovigilance could be strengthened by integrating tracking to link community referrals with health facility responses, currently tracking is manual. Although it increased costs, the technical support model improved after empowering district-level monitoring and evaluation officers to handle frontline technical troubleshooting. Establishing a frequently asked questions document and incorporating a technical support package within trainings enhanced troubleshooting efficiency and improved field teams’ ability to resolve issues independently.

### Supervision

A hierarchical supervision model improved accountability and performance. Rwanda’s HPV programme showed supervisor’s visibility of data improved oversight and outcomes (15). Updating SALAMA supervisor profiles would enable better performance tracking.

### Monitoring and evaluation

Real-time dashboards improved supervision, response speed and resource allocation, reducing stock shortages and enhancing data accuracy. However, challenges including absentee household tracking and incomplete supervision reporting persisted. Enhancing dashboard functionalities to include supervision reports, validation indicators and absentee household monitoring could improve monitoring and evaluation effectiveness [14].

### Data review and use

Digital approaches can improve campaign data use in decision-making. However, data completeness and timely data synchronization are paramount. Ensuring reliable power sources and connectivity are necessary to strengthen timely data entry and synchronization.

### WHO digital health intervention classification framework and SALAMA functionalities

SALAMA’s integration into the 2023–2024 SMC campaign can be analyzed using the WHO Digital Health Intervention Classification Framework to highlight strengths and areas for improvement.

#### Person

SALAMA lacked functionality to send SMS reminders to caregivers to administer SPAQ on Days 2 and 3, or post-referral visits. Although Day 2 and 3 adherence was high (~99%), this likely reflected strong counselling on Day 1, rather than ongoing engagement. Incorporating targeted communication could support adherence and follow-up.

#### Provider decision support

SALAMA validated age-based inclusion criteria and ensured correct SPAQ dosing, reducing errors previously observed in paper-based systems. However, SALAMA did not support structured exclusion workflows for fever or adverse events, which remained manual. The application should guide users through eligibility and referral steps. SALAMA also lacked functionality to detect duplicate registrations or multiple SPAQ administrations within a single cycle and did not identify fixed-point distributions where a door-to-door campaign was planned. These limitations highlight the need for more advanced decision-support features to improve consistency, reduce errors and better align implementation with protocols.

#### Provider-to-provider communication

SALAMA enabled real-time data sharing for key performance indicators across different levels, which facilitated responsive supervision and contributed to 92% Day 1 DOT adherence. However, SALAMA lacked local-level data sharing between community distributor supervisors and health facility coordinators, limiting local coordination. Pharmacovigilance tracking was also limited, preventing effective referral loop closure and effective adverse event management.

#### Supply chain management

SALAMA supported real-time stock entry and inventory tracking through dashboards, reducing stock-out risks. However, inconsistent use limited the tool’s potential.

#### Public health event notification

SALAMA enabled timely reporting of adverse events and SPAQ wastage, allowing supervisors to respond quickly.

#### Data services

SALAMA supported data collection, management and use, by structuring data collection, aggregation at district and provincial levels and visualization through real-time dashboards. This improved supervisors’ ability to identify low-performing districts and tailor mentorship. SALAMA improved data quality by enforcing mandatory fields and validating age-appropriate dosing. However, it failed to detect duplicates and did not enforce completeness, which meant some eligible children were registered but did not receive SPAQ doses without triggering any system alerts, as highlighted in end-of-round survey findings.

#### Data storage and transmission

SALAMA used secure electronic storage and standardized data entry fields to promote consistency and protect privacy. Data transmission and synchronization worked offline, later syncing to central servers, but challenges such as synchronization failures affected data coverage. These issues highlight the need for strong offline-first design, robust error handling and ongoing user training.

While SALAMA significantly strengthened routine campaign monitoring, supervision, stock management, and real-time decision-making, key gaps remain in communication, decision-support, pharmacovigilance tracking and data validation. Addressing these gaps will improve effectiveness and ensure alignment with WHO’s digital health best practices.

### Limitations

This work describes experience from implementation in Nampula Province, which may limit generalizability to other regions with different health system capacities or epidemiological contexts. The analysis is descriptive and observational, using routine administrative data and end-of-round survey results, without a control group or counterfactual design to rigorously measure impact. Administrative data may contain reporting errors or overestimation, and surveys data offers cross-sectional snapshots that may not capture the full picture. Some indicators, such as caregiver-reported adherence on Days 2 and 3, rely on self-reported data which may overstate actual adherence levels. Furthermore, systematic qualitative data from frontline health workers, supervisors or caregivers is not included, limiting insights into user experience or context-specific barriers. Cost, sustainability of the intervention and integration and interoperability with other national health information systems are outside the scope of this manuscript.

## CONCLUSION

Digitalizing the 2023–2024 SMC campaign improved operational efficiency, data-driven decision-making, supervision and data quality. Strong leadership and real-time monitoring enhanced campaign outcomes, however technical limitations, digital literacy gaps and connectivity issues remain. Addressing these through improved infrastructure, expanded training and strengthened data linkages will further strengthen future campaigns. These findings highlight how digital tools can improve campaign efficiency and effectiveness. Continued investment in digital health solutions and cross-sector collaboration are essential to ensure sustainability of SMC digitalization.

## Data Availability

No new data were generated or analysed in support of this publication. The data included in this article are from Seasonal Malaria Chemoprevention implementation round in 2023–2024 and can be shared on request to the corresponding author with permission of all parties.
